# Multiple Myeloma, Misdiagnosed As Somatic Symptom Disorder: A Case Report

**DOI:** 10.3389/fpsyt.2018.00557

**Published:** 2018-10-31

**Authors:** Jiashu Yao, Danmei Lv, Wei Chen

**Affiliations:** ^1^Department of Psychiatry, Sir Run Run Shaw Hospital, Hangzhou, China; ^2^School of Medcine, Zhejiang University, Hangzhou, China; ^3^Department of Psychiatry, Sir Run Run Shaw Hospital, Hangzhou, China

**Keywords:** misdiagnosis, somatic symptom disorder, somatic symptom and related disorders, multiple myeloma, case report

## Abstract

Here we report on a case of a 57-year-old woman with pain and discomfort in multiple sites of upper body who was diagnosed as somatic symptom disorder after completing a partial examinations of relevant parts which turned out to be negative. Finished imageological examinations of all painful parts, she was eventually diagnosed with multiple myeloma after 6-month being misdiagnosed as somatic symptom disorder. This case highlights the importance of completing imageological examinations of all the painful parts of the patient to exclude the possibility of multiple myeloma especially when symptoms are associated with objective signs and treatment has been ineffective; and it is as well as significant to notice characteristics of symptoms and to pay excessive attention directed toward the symptoms in the diagnosis of somatic symptom disorder.

## Introduction

Multiple myeloma (MM) is characterized by the neoplastic proliferation of immunoglobulin-producing plasma cells. Common presentations include anemia, bone pain, elevated creatinine or serum protein, fatigue, and hypercalcemia. Bone pain, particularly in the back or chest, and less often in the extremities, is present at the time of diagnosis in ~60% of patients (1). Imaging is a key part of the evaluation of all patients with suspected MM. If a patient with multiple myeloma initially presents with only bone pain but is not detected by imaging examination, it is probable to be diagnosed and treated as somatic symptom disorder, then to cause poor prognosis.

Here we report on a case in which a patient with MM was misdiagnosed as somatic symptom disorder (SSD). Written informed consent was obtained from this patient for the publication of this case report.

## Case report

### History of present illness

On January 4th, 2018, a 57-year-old woman was hospitalized in the department of Psychiatry, Sir Run Run Shaw Hospital because of pain and acid bilge in multiple sites of her upper body for more than 1 year. Over a year ago, the patient started feeling pain and discomfort in the upper left abdomen, and the pain got worse when coughing but with no other discomfort. Two months later, the upper left abdomen pain and acid bilge extended to the front chest, back, abdomen, and upper limbs. The symptoms persisted for months, and aggravated when changing body posture. Test results including cervical MRI, chest CT, abdominal B ultrasound of upper abdomen in a local hospital showed no abnormalities. Treated with Chinese medicine for more than 3 months, there was no significant improvement. About 6 months ago, the patient came to our hospital, expressing the symptoms above and worries about them, with weight loss of about 1–1.5 kg, but denying continuous depression, anxiety, and other symptoms (the score of 24 items of Hamilton Rating Scale for Depression was 12, and Hamilton Anxiety Rating Scale score was 11), and was diagnosed as “somatic symptom disorder.” After 4 months of treatment with 60 mg of duloxetine enteric-coated capsules twice daily and hypnotic drugs, the symptoms were obviously alleviated but not completely relieved and there was a significant weight loss of about 5 kg. Therefore, medication was adjusted to escitalopram tablets 20 mg once daily. Two months later, the patient felt no further improvement.

### Medical history

With hypertension history of more than 10 years, the patient claimed that it's not necessary for her to take any antihypertensive drugs to control blood pressure in recent 1 year. She had bronchitis for 12 years but no medicine was needed. She denied any history of diabetes, heart disease and other diseases and claimed there was no history of surgery and trauma. Also, the patient denied long-term chemical substances, drug or poison exposure history and had no history of smoking and drinking alcohol.

### Work-up and follow-up in the hospital

**Figure 1:**
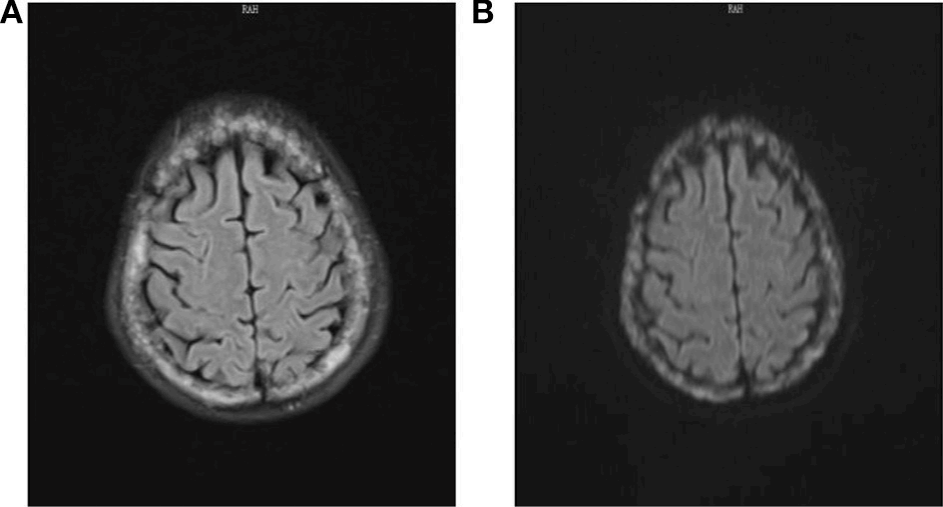
FLAIR **(A)** and DWI **(B)** sequences of cranial MRI showed diffuse thickening of the skull and increasing signal intensity.

After admission, due to long-term poor efficacy of the patient, we re-evaluated the patient's physical condition to rule out organic diseases. However, through Blood routine, Blood Biochemistry, Stool Routine, Urine Routine, Chest Film, Electrocardiogram, and so on, no specific abnormality was found. We found that the patient's tumor marker CA-153 was 61.2 U/mL (< 25.00 U/mL) and ferritin was 198.70 ug/L (13.00–150.00 ug/L), with no specificity. There was another finding of patient suffering from cholecystitis and gallstones through abdominal ultrasound examination; however, the surgeon suggested that it could not explain the patient's symptoms. When perfecting cranial MRI, we unexpectedly discovered below result: diffuse thickening of the skull and increasing signal intensity. Metastasis? Multiple myeloma? (Figure 1).

**Figure 2:**
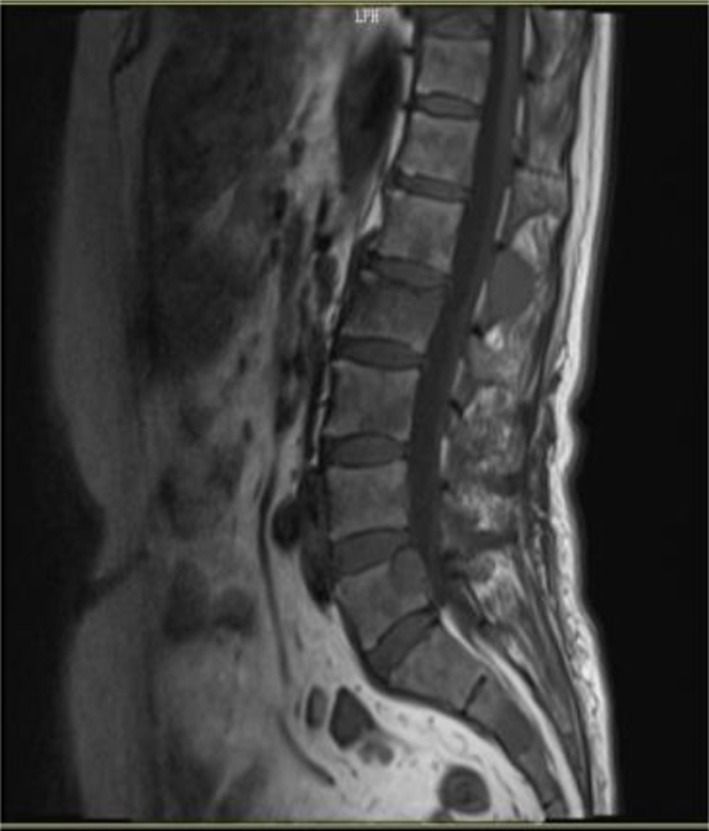
Lumbar MRI prompted lumbar vertebra multiple bone changes.

**Figure 3:**
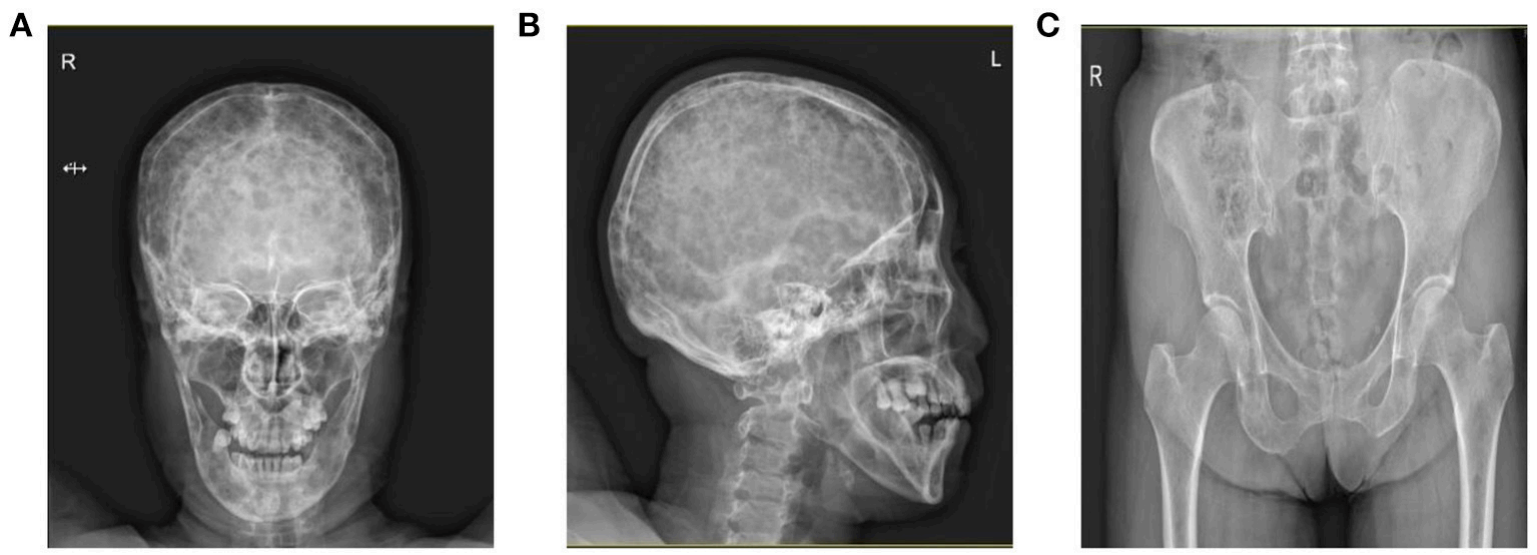
Skull X-ray prompted that the skull and maxillofacial bone **(A,B)** were found to have diffuse worm-like low-density bone destruction and there was no obvious hardening at the edge. **(C)** Pelvis X-ray showed small and low-density bone destruction zone in the pelvis and proximal femur.

And lumbar MRI prompted lumbar vertebra, attachment and right iliac bone multiple bone changes, multiple myeloma? Transfer? (Figure 2). Skull and pelvis plain radiographs prompted skull, maxillofacial bone, pelvis, and femoral bone changes, multiple myeloma? Transfer? (Figure 3).

**Figure 4:**
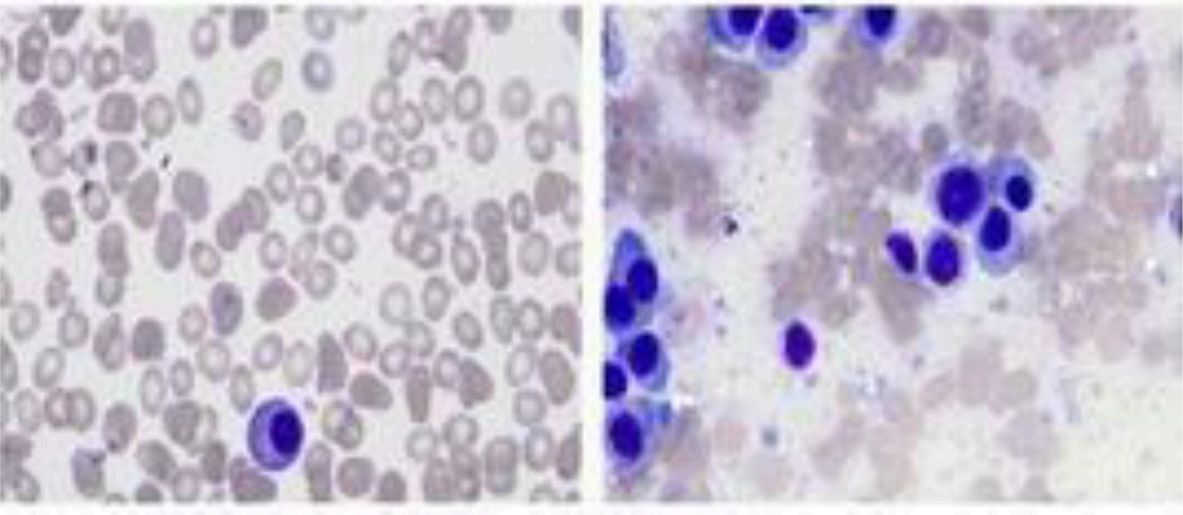
Bone marrow considered multiple myeloma, suggesting a significant increase in the proportion of plasma cells and a small amount of naive plasma cells.

After perfecting corresponding blood examination, the patient eventually underwent bone marrow aspiration and the results suggested that the patient was suffered from multiple myeloma (Figure 4). The patient was finally referred to the hematology department and received appropriate treatment.

### Discussion

Here we report on a case of a 57-year-old woman with pain and discomfort in multiple sites of upper body who was diagnosed as somatic symptom disorder after completing a partial examinations of relevant parts which turned out to be negative. Finished imageological examinations of all painful parts, she was eventually diagnosed with multiple myeloma after 6-month being misdiagnosed as somatic symptom disorder. This case highlights the importance of completing imageological examinations of all the painful parts of the patient to exclude the possibility of multiple myeloma especially when symptoms are associated with objective signs and treatment has been ineffective; and it is as well as significant to notice characteristics of symptoms and to pay excessive attention directed toward the symptoms in the diagnosis of somatic symptom disorder.

MM is a disease which is characterized by the neoplastic proliferation of immunoglobulin-producing plasma cells. Most patients with MM present with signs or symptoms related to the infiltration of plasma cells into the bone or other organs or to kidney damage from excess light chains. MM accounts for ~1–2% of all cancers and slightly more than 17% of hematologic malignancies (2). Worldwide, there are ~154,000 cases and 101,000 deaths per year attributed to MM (3). MM is also slightly more frequent in men than in women (~1.4:1). The risk of developing MM increases with body mass index (4, 5). MM is a disease of older adults. The median age at diagnosis is 66 years; only 10 and 2% of patients are younger than 50 and 40 years, respectively (1, 6).

Most patients with MM present with signs or symptoms related to the infiltration of plasma cells into the bone or other organs or to kidney damage from excess light chains. As an example, a retrospective analysis of 1,027 sequential patients diagnosed with MM at a single institution found the following symptoms and signs at presentation: Anemia-73%, Bone pain-58%, Elevated creatinine-48%, Fatigue/generalized weakness-32%, Hypercalcemia-28%, Weight loss-24%, one-half of whom had lost ≥9 kg (1).

In American Psychiatric Association's Diagnostic and Statistical Manual, Fifth Edition (DSM-5) (7), SSD is characterized by one or more somatic symptoms that are accompanied by excessive thoughts, feelings, and/or behaviors related to the somatic symptoms. It is estimated the prevalence in the general population is 4% (8, 9) and that among primary care patients is 17% (8, 10). An analysis of individual patient data from nine community studies (total *n* > 28,000) found that the most frequent burdensome symptom was pain (11). SSD is not defined by the number of distressful physical symptoms that are present; however, patients who complain about multiple symptoms are more likely to have the disorder. In this case, the patient had a number of pains and acid bilge in multiple locations which are typically present in somatic symptom disorder, with no other symptoms of MM, for instance, anemia, elevated creatinine, fatigue/generalized weakness, hypercalcemia. These enhance the possibilities of misdiagnosing MM as SSD. The percentage of underlying somatic diseases in patients previously diagnosed with SSD is relatively small but unneglectable. A meta-analysis (12) reviewed six diagnostic evaluation studies (total *N* = 1,804 patients), 16 follow-up studies (total *N* = 2,440 patients), and the percentage of misdiagnosis with SSD was 8.8% (95% CI 1.0–22.2, *p* = 0.007) in diagnostic evaluation studies, 0.5% (95% CI 0.01–1.5, *p* = 0.03) in follow-up studies, while the correct diagnosis shall be diabetes mellitus, duodenal ulcer, Crohn's disease, polymyalgia rheumatica, carcinoma, herniated disc, and so on.

Imaging is a key part of the evaluation of all patients with suspected MM. In this case, we found some related negative imaging test results like cervical MRI, chest CT, abdominal B ultrasound of upper abdomen from another hospital, however, neglected to do examinations of other important parts where the patient reported discomfort, such a lumbar and pelvis imageological examinations. Pain and acid bilge in multiple sites are usually associated with musculoskeletal and nervous system disease, and MRI is the best imaging choice for the early stage of these diseases. In the diagnosis procedure of SSD, thorough physical examination, laboratory tests and imageological examinations are necessary to help clinicians and patients build confidence and ensure that no important diagnosis will be missed (13–15). Moreover, criteria for selective use of tests include objective signs rather than the volume of the concerns expressed by the patient, the presence of complex symptoms, and persistence of symptoms (16). For instance, in our case, the pain of the patient aggravates when the body posture is changed or coughing. This characteristic probably points to a physical disease which is ignored during the early processes of out-patient treatment.

SSD patients always have excessive thoughts, feelings, or behaviors associated with the somatic symptoms. The patient was also anxious because of her symptoms which now we can consider it as healthy anxiety. Clinicians taking a history should determine whether somatic symptoms trigger healthy anxiety or not, in addition, determine whether the patient manifests persistent thoughts and anxiety related to the somatic symptoms, and whether the patient devotes excessive time and energy to the somatic symptoms (17–19). In International Classification of Disease-10 (ICD-10) (20) which is currently widely used in the world, somatoform disorders are defined on the basis of failure to find physical causes rather than the presence of definite psychological and behavioral features. The notion of taking medically unexplained symptoms as the defining feature of ICD-10 somatoform disorders creates a major hindrance to the clinical utility of the diagnosis. There is evidence that the decision about whether symptoms are medically unexplained is unreliable and lacks validity. The inherent dualism in the notion of a lack of medical explanation for somatic symptoms that are cross-sectionally assessed is simplistic and ultimately unhelpful to patent care (21). In ICD-11 (22), excessive attention directed toward the symptoms is highlighted in the diagnosis of Bodily distress disorder.

There is evidence that antidepressants are effective for SSD (23, 24). However, SNRIs could relieve pain by inhibiting reuptake of serotonin and norepinephrine, and suppressing painful sensation uploading regardless of physical disease or psychiatric disorders. Therefore, pain of the patient was obviously alleviated after 4 months of treatment with 60 mg of duloxetine enteric-coated capsules twice daily. This phenomenon also could perplex the revision of the diagnosis.

This case indicates that imageological examinations of all the painful parts of the patient should be completed to exclude the possibility of MM, especially of those whose symptoms are associated with objective signs and treatment has been ineffective. Furthermore, diagnosis of SSD requires not only the elimination of somatic diseases, but also excessive thoughts, feelings, or behaviors associated with the somatic symptoms.

## Ethics statement

This patient and his family provided informed consent for this case report.

## Author contributions

JY wrote the introduction and discussion, DL wrote the case report. WC guided the writing.

### Conflict of interest statement

The authors declare that the research was conducted in the absence of any commercial or financial relationships that could be construed as a potential conflict of interest.
